# The win ratio method: a novel hierarchical endpoint for pneumonia trials in patients on mechanical ventilation

**DOI:** 10.1186/cc13450

**Published:** 2014-03-17

**Authors:** A Montgomery, T Abuan, M Kollef

**Affiliations:** 1 Cardeas Pharma, Seattle, WA, USA; 2 Washington University, St Louis, MO, USA

## Introduction

Development of novel antibiotics for VAP is hampered by the need for large phase 3 trials with mortality endpoints. With allcause mortality rates decreasing, the large sample size for these trials, in particular for non-inferiority trials, has become a barrier to rapid drug development. Recently, a hierarchical approach to defining a composite endpoint for CV trials (win ratio method) has been described [1].

## Methods

We have adapted this approach in an ongoing superiority trial (http://Clinicaltrials.gov NCT01969799) comparing adjuvant use of a combination of fosfomycin and amikacin aerosol delivered by the PARI eFlow^® ^Inline nebulizer in patients with Gram-negative pneumonia who are on mechanical ventilation. Both groups are receiving standard- of-care i.v. antibiotics. Patients from the active and placebo groups are matched in pairs based on presence of MDR Gram-negative bacteria, and disease severity by APACHE II score. Each pair has a winner and a loser, or is a draw. The pair is first compared on mortality; if no difference, then ventilator-free days (VFD) are compared. If the outcomes are the same for both endpoints, a draw results. Active versus placebo groups will then be compared using the win ratio, defined as the number of pairs in which the active group was the winner divided by the number of pairs that did not result in a draw. We examined sample size and power characteristics of the win ratio endpoint using trial simulations.

## Results

Assuming a 15% 28-day mortality rate in the active arm and 20% in the control arm, to have 80% power with a two-tailed 0.05-level test for mortality would require 906 subjects per arm. Under the same assumptions with a difference in mean VFDs of 3 favoring the active arm (with common SD of 6), 130 subjects per arm provides 80% power. In approximately 32% of simulations, the win ratio result for each pair was determined by mortality. These simulation results assumed results within pairs were uncorrelated. If a positive correlation for each endpoint within pairs is assumed, power for the win ratio endpoint increases.

## Conclusion

The win ratio method is both clinically meaningful and straightforward to explain. This method could provide a new approach to powering both superiority and non-inferiority trials of novel antibiotics. In particular, this method allows for well-powered phase 2 trials, and potentially decreases the required size of phase 3 trials.
